# Paradoxically lowered oxygen isotopes of hydrothermally altered minerals by an evolved magmatic water

**DOI:** 10.1038/s41598-022-19921-y

**Published:** 2022-09-28

**Authors:** Chun-Sheng Wei, Zi-Fu Zhao

**Affiliations:** CAS Key Laboratory of Crust-Mantle Materials and Environments, School of Earth and Space Sciences, University of Science and Technology of China, Hefei, 230026 China

**Keywords:** Geochemistry, Mineralogy, Petrology

## Abstract

It has been well known that the influxing meteoric water can hydrothermally lower oxygen and hydrogen isotopes of rocks and/or minerals during continental magmatic or metamorphic processes in certain appropriate cases. Its opposite, however, is not implicitly true and needs independent testing. In terms of a novel procedure recently proposed for dealing with thermodynamic re-equilibration of oxygen isotopes between constituent minerals and water from fossil hydrothermal systems, the initial oxygen isotopes of water (${\updelta }^{18}{\text{O}}_{\text{W}}^{\text{i}}$) are theoretically inverted from the early Cretaceous post-collisional granitoids and Triassic gneissic country rock across the Dabie orogen in central-eastern China. Despite ancient meteoric waters with low ${\updelta }^{18}{\text{O}}_{\text{W}}^{\text{i}}$ value down to − 11.01 ± 0.43‰ (one standard deviation, 1SD), oxygen isotopes of hydrothermally altered rock-forming minerals from a granitoid were unexpectedly but concurrently lowered by an evolved magmatic water with mildly high ${\updelta }^{18}{\text{O}}_{\text{W}}^{\text{i}}$ value of 2.81 ± 0.05‰ at 375 °C with a water/rock (W/R)_c_ ratio of 1.78 ± 0.20 for the closed system. The lifetime of fossil hydrothermal systems studied herein is kinetically constrained to no more than 1.2 million years (Myr) via surface-reaction oxygen exchange in the late-stage of continental magmatism or metamorphism. Thereby, caution should be paid when lowered oxygen isotopes of hydrothermally altered rocks and/or minerals were intuitively and/or empirically inferred from the external infiltration of the purely meteoric water with a low ${\updelta }^{18}{\text{O}}_{\text{W}}^{\text{i}}$ value alone.

## Introduction

Among major natural water reservoirs (i.e., oceanic, meteoric, magmatic and metamorphic water, etc.) feeding the modern geothermal and/or ancient hydrothermal systems across the Earth, there is no doubt that both δ^18^O and δ^2^H values of the meteoric water precipitated at the mid- to high-latitude are less than 0‰ on the global scale^1–8^. Because meteoric water with low ^18^O/^16^O and/or ^2^H/^1^H ratios is usually far from thermodynamic equilibrium with most rocks of interest, the meteoric hydrothermal alteration was thus proposed as a favourable and/or effective process for the evident lowering of oxygen and hydrogen isotopes documented worldwide (see Refs.^9–15^ and extensive references therein). Thereby, hydrothermally altered rocks and/or minerals with lowered oxygen and hydrogen isotopes were exclusively concluded being imprinted by the meteoric water within continental settings. These insights, however, still need a more integrated verification in further details.

While conventional straightforward modellings have been attempted numerous times over the past decades^9–11^, the lowering of oxygen and hydrogen isotopes for hydrothermally altered rocks is actually regulated by many physicochemical boundary conditions like the initial isotopes of water and rock, W/R ratio, alteration temperature as well as the nature of hydrothermal systems (closed vs. open). Most of the variables, however, were arbitrarily presumed or empirically estimated but not quantitatively constrained by previous studies instead. Thus, these uncertainties seriously limit the quantification for the lowering of oxygen and hydrogen isotopes of hydrothermally altered rocks.

**Figure 1:**
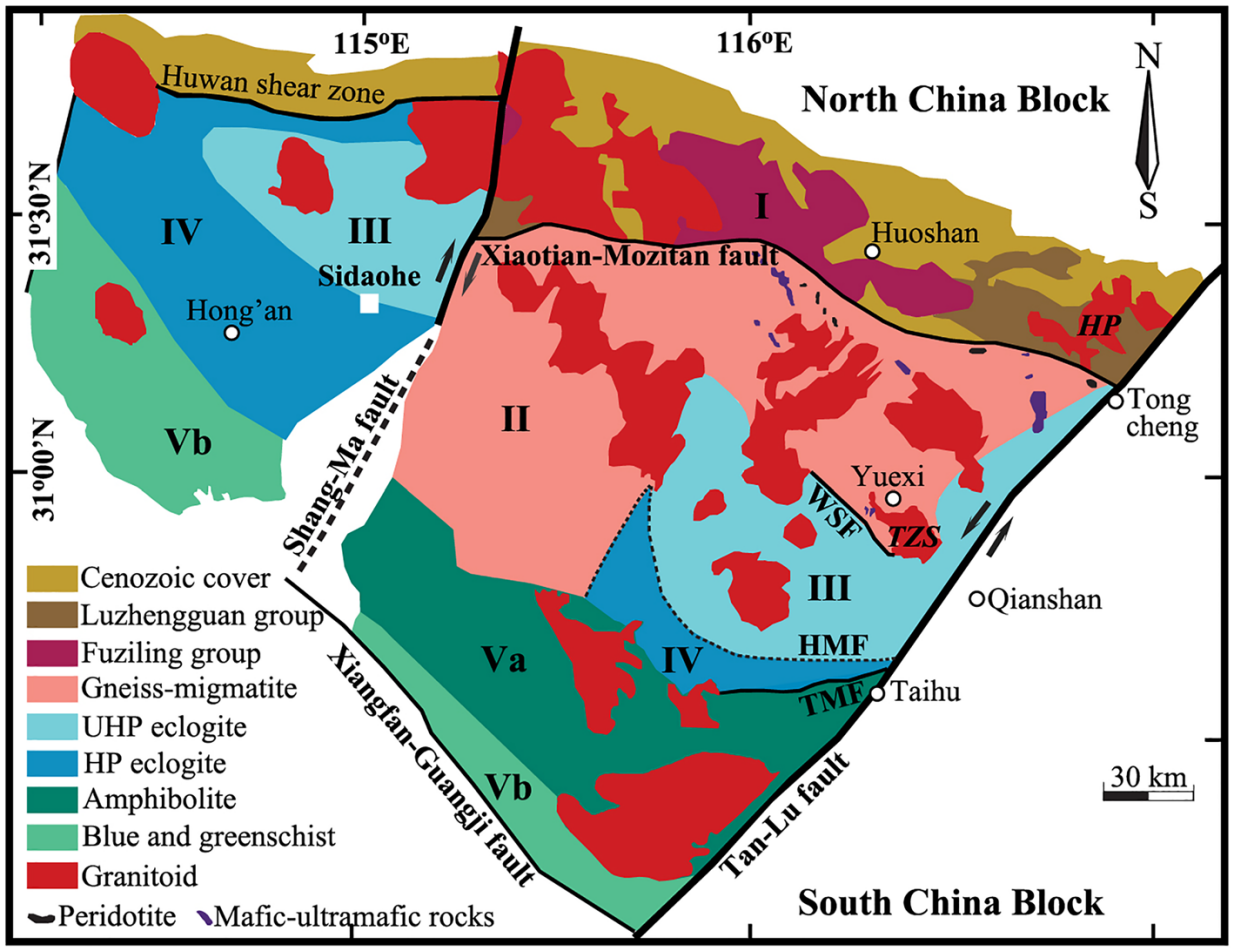
Geological sketch of the Dabie orogen in central-eastern China modified from Refs.^18–20^. In terms of field relation and lithological assemblage, geologic units bounded with faults were divided. Traditionally, the western portion beyond the Shang-Ma fault was termed Hong’an (or Xinxian) Block. The Dabie Block (DBB) is bounded by the Shang-Ma fault in the west and the Tan-Lu fault in the east. From north to south, the DBB was further subdivided into five belts: I. the Northern Huaiyang volcanic-sedimentary belt (flysch series); II. the Northern Dabie gneissic and migmatitic belt; III. the Central Dabie ultrahigh pressure (UHP) metamorphic belt; IV. the Southern-central Dabie high pressure metamorphic belt; and V. the Southern Dabie intermediate- to low-grade metamorphic belt, respectively. *WSF* Wuhe-Shuihou fault, *HMF* Hualiangting-Mituo fault, and *TMF* Taihu-Mamiao fault. Bold italic capital letters denote the abbreviations of studied plutons, other details refer to Table S1. This figure was generated with Adobe Photoshop CS3 Extended version 10.0 (https://www.adobe.com/cn/products/photoshop.html).

It has been well known that hydrothermal systems are essentially driven by natural heat engines, the lowering of oxygen and hydrogen isotopes for altered rocks accompanying magmatism and/or metamorphism is thus more realistic for thermodynamic and kinetic considerations. Since magmatic and metamorphic rocks are widespread across the Dabie orogen in central-eastern China (Fig. 1 and details see “Methods” section), these enable the quantification for the lowering of oxygen and hydrogen isotopes from hydrothermally altered rocks more possible. On the other hand, while both hydrogen and oxygen isotopes can potentially be applied to trace the fossil hydrothermal systems, the more reliable fractionation relationship and higher precision of analysis make oxygen isotopes more robust and/or ideal^12–15^. Furthermore, it is worthwhile pointing out that more faithful clues can be quantitatively decoded from the hydrothermally mineral pairs rather than individual whole-rocks. Because concurrently lowered oxygen isotopes among constituent minerals were analytically observed in this study, this enhances theoretical inversion of the ${\updelta }^{18}{\text{O}}_{\text{W}}^{\text{i}}$ values more practicable^16,17^.

## Results

**Figure 2:**
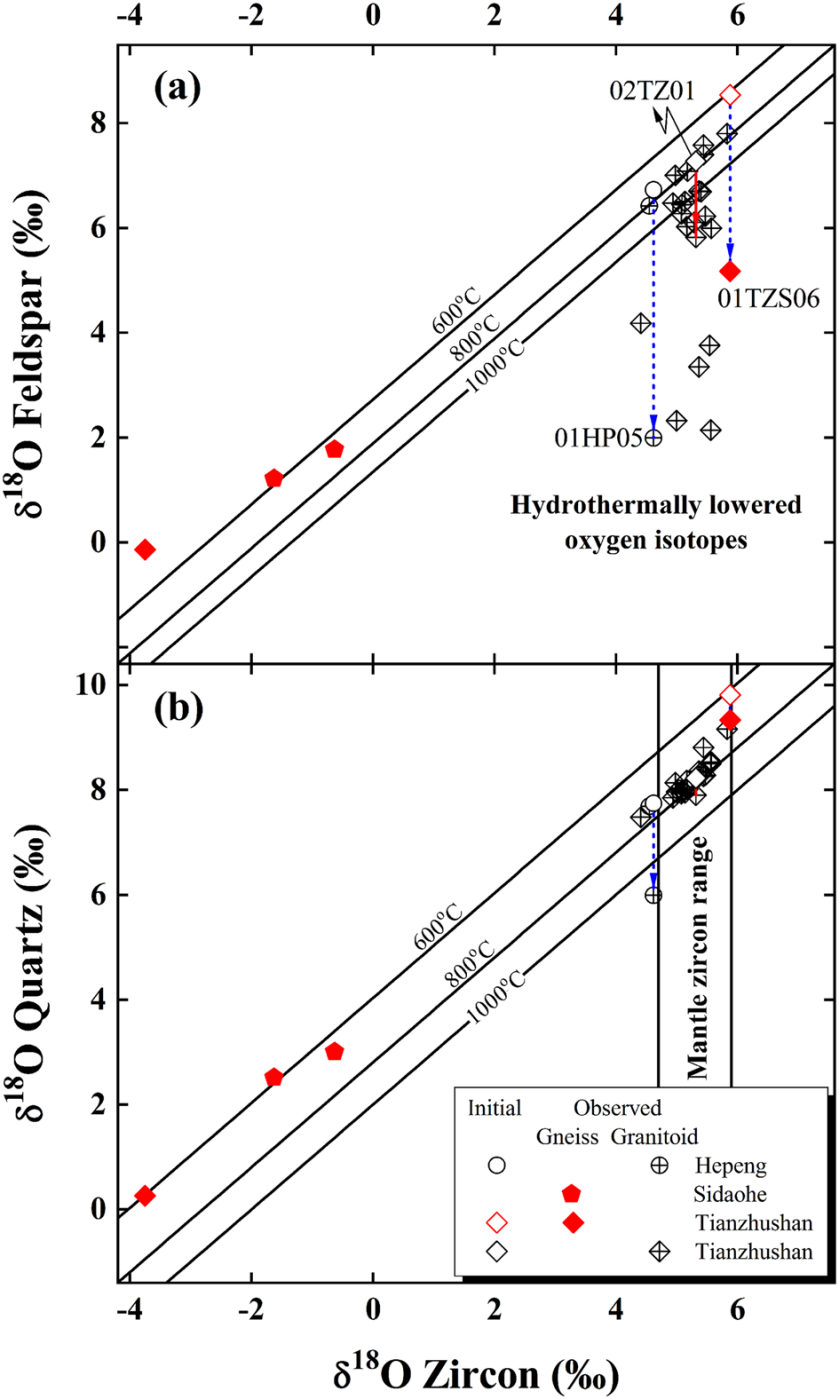
Diagrams of zircon vs. alkali feldspar (**a**) and quartz δ^18^O values (**b**) for the granitoids and gneisses from the Dabie orogen. Lines labelled with temperatures are isotherms after Ref.^21^, and two vertical solid lines in (**b**) denote the variability of mantle zircon δ^18^O values (5.3 ± 0.6‰) for comparison^22^. Arrowed lines denote samples theoretically inverted in this study. The observed and initial oxygen isotopes of constituent minerals refer to Table S1 and Table 1, respectively. The error bar is omitted for clarity herein.

It can be seen that zircon δ^18^O values of the early Cretaceous post-collisional granitoids in this study cluster around 5.16 ± 0.38‰ (n = 25, details see “Methods” section and Table S1) and overlap with mantle zircon (Fig. 2). In contrast, zircon oxygen isotopes of the Triassic gneisses scatter from − 3.78 to 5.88‰ herein. Isotopically, the granitoids with uniform zircon δ^18^O values cannot dominantly originate from those heterogeneous gneisses, which are almost sevenfold larger than zircon δ^18^O variability of the granitoids (9.66 vs. 1.42‰).

Steep departure from isotherms downwards and evident disequilibrium with zircon oxygen isotopes, however, appear for some alkali feldspar δ^18^O values of the studied granitoids and gneissic country rock (Fig. 2a). Further data examinations show that quartz oxygen isotopes are concurrently lowered with alkali feldspar for the labelled samples although equilibrium fractionations are well preserved by most of available zircon and quartz oxygen isotopes (Fig. 2b).

Because ^18^O-depleted metamorphic rocks were regionally documented across the Dabie orogen^16,23–25^, oxygen isotopes of the studied granitoids could be lowered through the magmatic assimilation by metamorphic country rocks (in particular gneisses in most cases). It can be seen that the normal zircon δ^18^O value of sample 02TZ01 is apparently overlapped with others from the Tianzhushan granitoid pluton (Table S1, Fig. 2), and its lowered oxygen isotopes of rock-forming minerals are independent of the gneissic county rocks with heterogeneous oxygen isotopes (samples 01TZS06 and 01TZS07 in Table S1). Moreover, magmatic temperatures of 780 ± 20 °C for the Tianzhushan pluton (see footnote of Table 1) are actually affiliated to the cold granitoid^26^. This seems less favourable for the assimilation by the gneissic county rocks because the latent heat is energetically limited with the crystallisation of a cold granitoid. While subtly low zircon δ^18^O values are indeed observed from the Hepeng granitoids (4.54 to 4.64‰ for samples 01HP04 and 01HP05 in Table S1, Fig. 2), their extreme homogeneity is fundamentally inconsistent with the progressive assimilating on the pluton scale. Similarly, magmatic temperatures lower than 800 °C for the Hepeng pluton (see footnote of Table 1) also restricted the potential assimilation during magma cooling processes. On the other hand, the country rocks intruded by the Hepeng pluton are volcanic-sedimentary rocks (Fig. 1), which are ^18^O-enriched rather than -depleted in most circumstances. In fact, these upper continental crusts themselves are too cold to substantially assimilate oxygen isotopes of the Hepeng granitoids.

**Table 1:** Parameters of theoretical inversion for ${\updelta }^{18}{\text{O}}_{\text{W}}^{\text{i}}$ values of the externally (E) infiltrated and internally (I) buffered water.

Sample number	${\updelta }^{18}{\text{O}}_{\text{Qtz}}^{\text{i}}$(‰)*	${\updelta }^{18}{\text{O}}_{\text{Ksp}}^{\text{i}}$(‰)*	T (°C)^†^	${\updelta }^{18}{\text{O}}_{\text{W}}^{\text{i}}$ (‰)^‡^
**Granitoid**				
01HP05	7.74 ± 0.02	6.72 ± 0.02	140 ± 5	− 11.01 ± 0.43 (E)
02TZ01	8.23 ± 0.10	7.28 ± 0.07	375	2.81 ± 0.05 (I)
**Gneiss**				
01TZS06	9.81 ± 0.15	8.54 ± 0.10	130 ± 5	− 8.52 ± 0.56 (E)
${\text{n}}_{\text{Water}}^{\text{O}}/{\text{n}}_{\text{mineral}}^{\text{O}}$^§^	1.68	1.93		

*Initial quartz (${\updelta }^{18}{\text{O}}_{\text{Qtz}}^{\text{i}}$) and alkali feldspar (${\updelta }^{18}{\text{O}}_{\text{Ksp}}^{\text{i}}$) oxygen isotopes are calculated with the observed zircon δ^18^O values (Table S1) at magmatic or metamorphic temperatures, respectively. The initial oxygen isotopes of sample 01HP05 are calculated at 740 ± 1 °C, which is retrieved from oxygen isotopes of quartz-zircon pair from sample 01HP04 and assumed a similar magmatic temperature on the pluton scale. The averaged magmatic temperature of 780 ± 20 °C from the Tianzhushan pluton, however, is adopted for sample 02TZ01. The metamorphic temperature of 610 ± 20 °C is bracketed through samples 00DB63, 00DB64 and 01TZS07 and a common thermal regime on the orogenic scale is assumed for sample 01TZS06.

^†^Re-equilibration temperatures are calculated with the observed oxygen isotopes of quartz-alkali feldspar pairs. Owing to the lack of repetitive measurements for sample 02TZ01 (Table S1), its 1SD of re-equilibration temperature is statistically not assigned.

^‡^Theoretically inverted from the open systems for samples 01HP05 and 01TZS06 whereas from the closed system for sample 02TZ01.

^§^Ratio of exchangeable oxygen content between water and an indicated mineral.

Owing to the complexity and sluggishness of metamorphic reactions, the lowered oxygen isotopes of gneissic country rocks studied herein could alternatively inherit from their protoliths with original non-equilibria. However, two gneisses from the Sidaohe in the Hong’an Block well maintain equilibrium fractionations between zircon and quartz as well as alkali feldspar oxygen isotopes (samples 00DB63, 00DB64 in Table S1, Fig. 2). Moreover, the equilibrium fractionation between zircon and quartz oxygen isotopes is also retained for sample 01TZS07 from gneissic country rock of the Tianzhushan pluton (Table S1, Fig. 2b). While their oxygen isotopes among constituent minerals are evidently varied, similar equilibrium fractionations were thermodynamically achieved for the above samples (particularly between zircon and quartz oxygen isotopes in Fig. 2b). Thereby, it is hardly to expect that original non-equilibria of oxygen isotopes were exceptionally inherited from their protoliths both for the less mobile quartz and reactive alkali feldspar for sample 01TZS06, which is spatially no more than 3 km apart from sample 01TZS07 (see GPS data in Table S1). Furthermore, previous studies showed that the inert Sm–Nd, Lu–Hf and U–Pb radiometric systems were reliably homogenised and/or reset during the Triassic metamorphism across the Dabie orogen^27–33^. Thus, it is less likely that the actively liable oxygen of rock-forming minerals could survive the continental deep subduction and be inherited from their protoliths.

Collectively, the concurrently lowered oxygen isotopes of quartz and alkali feldspar in this study are best attributed to hydrothermal alteration during the post-magmatic and/or exhumation processes of the retrograde metamorphism across the Dabie orogen. This is in a good agreement with the less high re-equilibration temperatures discussed below.

### Theoretical inversion of meteoric water with low ${\updelta }^{18}{\text{O}}_{\text{W}}^{\text{i}}$ values

**Figure 3:**
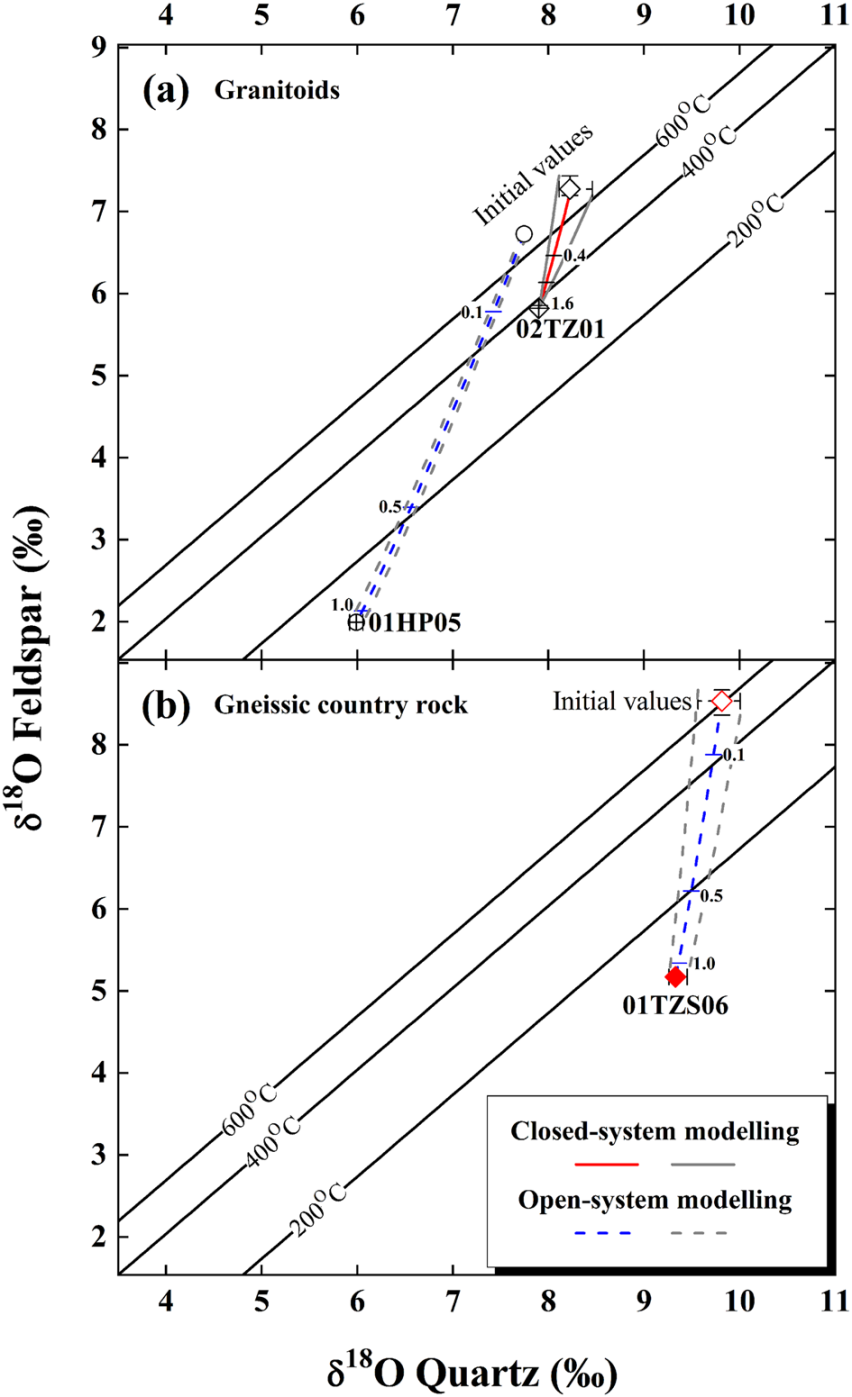
Diagrams of quartz vs. alkali feldspar δ^18^O values for the granitoids (**a**) and gneissic country rock (**b**) from the Dabie orogen. The curves with gray envelopes denote the maximum variability for the concurrently lowered oxygen isotopes of rock-forming minerals, re-equilibrated with the externally infiltrated meteoric water (blue) or internally buffered yet evolved magmatic water (red), respectively, throughout this study. Small ticks with numbers are W/R ratios. Other details refer to Figs. S1–S3 and Fig. 2.

It can be seen that oxygen isotopes of quartz were thermodynamically re-equilibrated with alkali feldspar at 140 ± 5 °C for a granitoid from the Hepeng pluton (sample 01HP05 in Table 1, Fig. 3a). In terms of procedures described in “Methods” section, the ${\updelta }^{18}{\text{O}}_{\text{W}}^{\text{i}}$ value is theoretically inverted as − 11.01 ± 0.43‰ for the open system, which is consistent with a meteoric water externally infiltrated into the studied granitoid. On the basis of parameters listed in Table 1, the currently lowered oxygen isotopes of hydrothermally altered rock-forming minerals are well reproduced (Fig. 3a). A minimum (W/R)_o_ ratio of 1.10 ± 0.02 is accordingly yielded. It is worthwhile pointing out that a systematically high (W/R)_c_ ratio is quantified if the closed system is adopted (arrowed solid vs. dashed vertical lines in Fig. S1).

For the gneissic country rock intruded by the Tianzhushan granitoid pluton, a re-equilibration temperature of 130 ± 5 °C is yielded (sample 01TZS06 in Table 1, Fig. 3b). The ${\updelta }^{18}{\text{O}}_{\text{W}}^{\text{i}}$ value of the meteoric water is thus theoretically inverted as − 8.52 ± 0.56‰ for the open system, and a (W/R)_o_ ratio of 1.19 ± 0.05 is constrained for the concurrently lowered oxygen isotopes of hydrothermally altered rock-forming minerals (Fig. S2, Fig. 3b).

While the low ${\updelta }^{18}{\text{O}}_{\text{W}}^{\text{i}}$ values of ancient meteoric water theoretically inverted above are varied across the Dabie orogen, they are in fact reasonable values compared to most mountainous terranes worldwide and their palaeo-environmental implications could be analogously exploited elsewhere^34–37^.

### Theoretical inversion of an evolved magmatic water with mildly high ${\updelta }^{18}{\text{O}}_{\text{W}}^{\text{i}}$ value

Compared to samples studied above, the concurrently lowered oxygen isotopes for quartz and alkali feldspar are less evident for a granitoid from the Tianzhushan pluton (labelled sample 02TZ01 in Fig. 2a). In contrast to 7.87 ± 0.27‰ of the primary magmatic water illustrated in the following Fig. 5a, a mildly high ${\updelta }^{18}{\text{O}}_{\text{W}}^{\text{i}}$ value of 2.81 ± 0.05‰ is unexpectedly but uniquely inverted at a re-equilibration temperature of 375 °C (Table 1, Fig. 3a). More interestingly, the observed oxygen isotopes of hydrothermally altered rock-forming minerals were paradoxically lowered by this evolved magmatic water with a (W/R)_c_ ratio of 1.78 ± 0.20 for the closed system (Fig. S3, Fig. 3a).

## Discussion

### The reliability of ${\updelta }^{18}{\text{O}}_{\text{W}}^{\text{i}}$ values

While ${\updelta }^{18}{\text{O}}_{\text{W}}^{\text{i}}$ values of ancient meteoric and evolved magmatic water are theoretically inverted from the concurrently lowered oxygen isotopes of hydrothermally altered rock-forming minerals in this study, their reliability needs to be further assessed. As shown in Eqs. (1) through (3) in “Methods” section, both re-equilibration temperature and initial oxygen isotopes of constituent minerals are prerequisites in order to theoretically invert the ${\updelta }^{18}{\text{O}}_{\text{W}}^{\text{i}}$ value. The re-equilibration temperatures are calculated with oxygen isotopes of quartz-alkali feldspar pairs observed for corresponding samples. The initial oxygen isotopes of rock-forming minerals, however, are constrained with oxygen isotopes of the inert zircon at magmatic or metamorphic temperatures. In these cases, the role of temperatures is evaluated to validate ${\updelta }^{18}{\text{O}}_{\text{W}}^{\text{i}}$ values theoretically inverted above.

#### Construction of the relationship between temperatures and ${\updelta }^{18}{\text{O}}_{\text{W}}^{\text{i}}$ values

**Figure 4:**
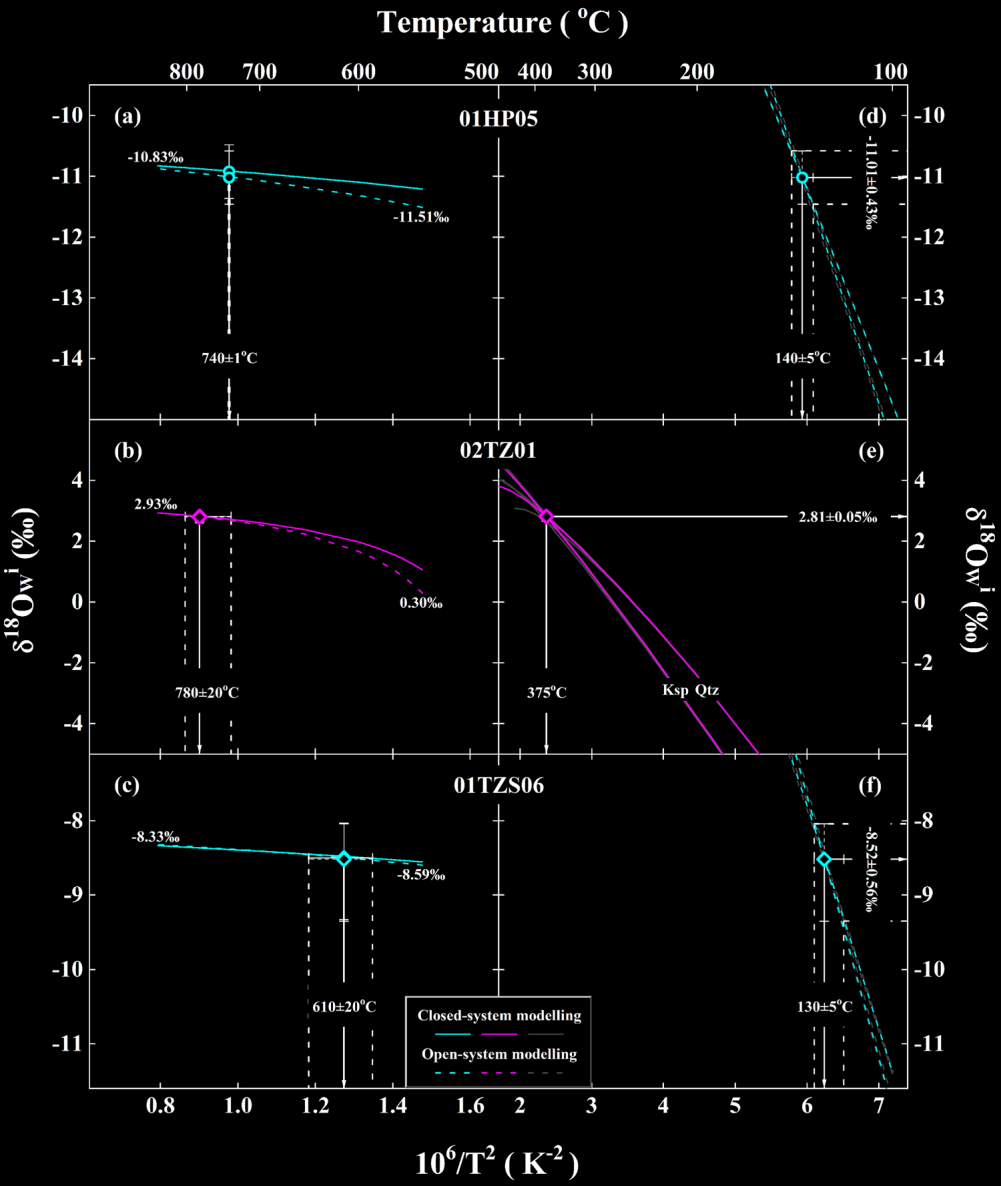
The relationship between temperatures and ${\updelta }^{18}{\text{O}}_{\text{W}}^{\text{i}}$ values theoretically inverted from the Dabie orogen. Curves with gray envelopes denote ${\updelta }^{18}{\text{O}}_{\text{W}}^{\text{i}}$ values theoretically inverted from the early Cretaceous post-collisional granitoids (samples 01HP05 and 02TZ01) and the Triassic gneissic country rock (sample 01TZS06), respectively. Arrowed vertical lines with envelopes illustrate the maximum variation of magmatic (**a,b**) and metamorphic (**c**) as well as re-equilibration temperatures (**d–f**) adopted for the studied samples. Symbol points with error bars denote the maximum variability of ${\updelta }^{18}{\text{O}}_{\text{W}}^{\text{i}}$ values and temperatures. Other details see text.

In order to verify the likely effects of re-equilibration temperature and magmatic or metamorphic temperatures on theoretical inversion of ${\updelta }^{18}{\text{O}}_{\text{W}}^{\text{i}}$ values for the meteoric and evolved magmatic water in this study, two strategies are applied to deal with these issues separately:

1. The mean re-equilibration temperature is fixed for each studied sample. Because the magmatic temperature ranging from 740 to 800 °C and metamorphic temperature of 610 ± 20 °C are adopted throughout this study (Table 1, Figs. 4a–c), their maximum variations are arbitrarily set from 550 to 850 °C. Then, the initial oxygen isotopes of rock-forming minerals are calculated with the observed zircon oxygen isotopes at a hypothetical temperature through Eq. (3). From the low- to high-end, five to seven temperature intervals are usually conducted in this study (e.g., 550, 600, 650,…, 850 °C). Substituting these new initial oxygen isotopes into Eqs. (1) and (2), a hypothetical ${\updelta }^{18}{\text{O}}_{\text{W}}^{\text{i}}$ value can be theoretically inverted for the closed system. Similar procedures can be applied to the open system. These results are thus illustrated as labelled curves in Figs. 4a–c.
2. Owing to the susceptibility of common rock-forming minerals to hydrothermal alteration (in particular feldspar), an apparent rather than true re-equilibration temperature could result under some situations. In order to test the potential influence of varied re-equilibration temperatures on theoretical inversion of ${\updelta }^{18}{\text{O}}_{\text{W}}^{\text{i}}$ values, the following procedures are carried out. First, the observed oxygen isotopes of alkali feldspar are fixed for each studied sample. Then, quartz δ^18^O values are reasonably adjusted to either higher or lower values (five to seven adjustments are adequate in most cases). A hypothetical re-equilibration temperature is accordingly calculated with the combination of the observed and adjusted oxygen isotopes. Substituting these values into Eqs. (1) and (2), a hypothetical ${\updelta }^{18}{\text{O}}_{\text{W}}^{\text{i}}$ value can be theoretically inverted for the closed system. Similar procedures can be applied to the open system and these results are illustrated as curves labelled with Qtz in Figs. 4d–f. When the observed oxygen isotopes of quartz are fixed and alkali feldspar δ^18^O values are adjustable, the resulting curves are labelled with Ksp. It is worthwhile pointing out that the mean magmatic or metamorphic temperatures with maximum variations are adopted for constructing curves with gray envelops in Figs. 4d–f.

#### The impact of magmatic or metamorphic temperatures on ${\updelta }^{18}{\text{O}}_{\text{W}}^{\text{i}}$ values

It can be seen that ${\updelta }^{18}{\text{O}}_{\text{W}}^{\text{i}}$ values gradually increase with higher magmatic or metamorphic temperatures (Figs. 4a–c). Moreover, a subtle increase of ${\updelta }^{18}{\text{O}}_{\text{W}}^{\text{i}}$ values is systematically inverted for the closed system and converged with those inverted for the open system (solid vs. dashed curves in Figs. 4a–c). With the labelled ${\updelta }^{18}{\text{O}}_{\text{W}}^{\text{i}}$ values, their maximum variability is generally less than 0.88‰ along with the temperatures varied for every 100 °C for all studied samples herein. On the other hand, since the actual variation of temperatures is much more limited (arrowed vertical lines with envelopes in Figs. 4a–c), the corresponding variability of ${\updelta }^{18}{\text{O}}_{\text{W}}^{\text{i}}$ values would be much less than the maximum variability of 0.88‰. For example, the 1SD of ± 0.05‰ is yielded for ${\updelta }^{18}{\text{O}}_{\text{W}}^{\text{i}}$ variability of sample 02TZ01 (Table 1, Fig. 4e), which is only dependent upon the varied magmatic temperatures. Nevertheless, these suggest that magmatic or metamorphic temperatures adopted in this study cannot significantly affect the variability of ${\updelta }^{18}{\text{O}}_{\text{W}}^{\text{i}}$ values and their reliability is thus guaranteed.

#### The influence of re-equilibration temperatures on ${\updelta }^{18}{\text{O}}_{\text{W}}^{\text{i}}$ values

While the impact of magmatic or metamorphic temperatures is limited, the variability of ${\updelta }^{18}{\text{O}}_{\text{W}}^{\text{i}}$ values is sufficiently evident (Table 1 and symbol points with error bars in Fig. 4). In this regard, the influence of re-equilibration temperatures is further assessed.

Compared with the limited impact by magmatic or metamorphic temperatures, the variability of ${\updelta }^{18}{\text{O}}_{\text{W}}^{\text{i}}$ values is more sensitive to re-equilibration temperatures. It can be seen that a large range of ${\updelta }^{18}{\text{O}}_{\text{W}}^{\text{i}}$ values is theoretically inverted with the varied re-equilibration temperatures (curves with gray envelopes in Figs. 4d–f). A cross point, however, appears for each studied sample. This means that both quartz and alkali feldspar oxygen isotopes were re-equilibrated with a unique water at the same temperature for individual samples, which just correspond to the ${\updelta }^{18}{\text{O}}_{\text{W}}^{\text{i}}$ values theoretically inverted with the concurrently lowered oxygen isotopes of hydrothermally altered quartz and alkali feldspar (Table 1 and symbol points with error bars in Figs. 4d–f). In this respect, it suggests that thermodynamic re-equilibration was attained and/or achieved at least between the studied rock-forming minerals and water and the ${\updelta }^{18}{\text{O}}_{\text{W}}^{\text{i}}$ values of meteoric and evolved magmatic water are therefore validated. Moreover, it is worthwhile pointing out that an evidently low ${\updelta }^{18}{\text{O}}_{\text{W}}^{\text{i}}$ value of − 11.01 ± 0.43‰ is theoretically inverted from sample 01HP05 although its re-equilibration temperature is similar to that of sample 01TZS06 (Table 1, Figs. 4d,f). This confidently enhances the reliability of ${\updelta }^{18}{\text{O}}_{\text{W}}^{\text{i}}$ values theoretically inverted in this study.

#### Assessment of the uncertainties of theoretical inversion

During theoretical inversion for ${\updelta}^{18}{\text{O}}_{\text{W}}^{\text{i}}$ values, there are at least three types of input variables required (i.e., ${\updelta }^{18}{\text{O}}_{\text{Ksp}}^{\text{f}}$ or ${\updelta }^{18}{\text{O}}_{\text{Qtz}}^{\text{f}}$ values, ${\updelta }^{18}{\text{O}}_{\text{Ksp}}^{\text{i}}$ or ${\updelta }^{18}{\text{O}}_{\text{Qtz}}^{\text{i}}$ values as well as ${{{\text{(}{\Delta}}^{18}{\text{O}}}_{\text{W}}^{\text{Ksp}}\text{)}}_{\text{r}}$ or ${{{\text{(}\Delta}^{18}{\text{O}}}_{\text{W}}^{\text{Qtz}}\text{)}}_{\text{r}}$ values in Eqs. (1) to (3)). Thus, their direct and/or indirect contribution to the uncertainties of theoretical inversion are assessed individually.

For the initial oxygen isotopes of rock-forming minerals, their uncertainties inherit and/or propagate from analytical error of zircon δ^18^O values and variability of magmatic or metamorphic temperatures adopted. While the analytical error of zircon δ^18^O values is the best for sample 01TZS06 among all of available data in this study (± 0.01‰ in Table S1), the uncertainties of initial oxygen isotopes of their rock-forming minerals are not correspondingly the smallest (Table 1, Fig. S2, Fig. 3b). In contrast, owing to the limited variation of magmatic temperatures (740 ± 1 °C) adopted, the smallest uncertainties of ± 0.02‰ are accordingly yielded for the initial oxygen isotopes of rock-forming minerals of sample 01HP05 (Table 1). In these cases, it suggests that the uncertainties of initial oxygen isotopes of rock-forming minerals are more sensitive to the variation of magmatic or metamorphic temperatures adopted. Furthermore, because oxygen isotope fractionations between quartz and zircon are systematically larger than those between alkali feldspar and zircon^21^, slightly evident uncertainties of initial oxygen isotopes for quartz accordingly appear (Table 1, Figs. S2, S3, Fig. 3).

The re-equilibration temperature is calculated with the observed oxygen isotopes between quartz and alkali feldspar throughout this study. Therefore, its uncertainty is dependent upon the analytical precision of constituent minerals. Owing to the comparable precision for quartz (0.10 vs. 0.11‰ in Table S1), a similar uncertainty of ± 5 °C is yielded for the re-equilibration temperatures of samples 01HP05 and 01TZS06 (Table 1, Figs. 4d,f).

The smallest uncertainty of ± 0.05‰ for ${\updelta}^{18}{\text{O}}_{\text{W}}^{\text{i}}$ values is yielded for sample 02TZ01 (Table 1), this is probably due to the least varaition of its re-equilibration temperature (Fig. 4e). On the contrary, the largest uncertainty of ± 0.56‰ occurs for sample 01TZS06 owing to its varied re-equilibration temperature (Table 1, Fig. 4f). While the variation of their re-equilibration temperatures is statistically comparable (± 5 °C), the uncertainty of ${\updelta }^{18}{\text{O}}_{\text{W}}^{\text{i}}$ values for sample 01TZS06 is relatively larger than that for sample 01HP05 (± 0.56 vs. ± 0.43‰ in Table 1, Figs. 4f,d). These could further result from the large uncertainties of initial oxygen isotopes of rock-forming minerals for sample 01TZS06 (Table 1, Fig. S2, Fig. 3b). Nonetheless, the uncertainties of ${\updelta }^{18}{\text{O}}_{\text{W}}^{\text{i}}$ values could be further improved and/or refined in the future with more precise re-equilibration temperatures as well as less varied magmatic or metamorphic temperatures adopted. The accuracy of ${\updelta }^{18}{\text{O}}_{\text{W}}^{\text{i}}$ values theoretically inverted in this study, however, is statistically robust.

While the limited variation of less than ± 0.05 occurs for the (W/R)_o_ ratios from the open system, a larger variation of (W/R)_c_ ratios from the closed system is systematically yielded (Table 2, Figs. S1–S3, Fig. 3). The variation of (W/R)_c_ ratios for sample 01TZS06 is statistically larger than that for sample 01HP05 with similar variation of re-equilibration temperatures for the closed  system (± 0.38 vs. ± 0.15 in Table 2). These could attribute to the distinctive uncertainties of initial oxygen isotopes of their rock-forming minerals (Table 1). Consequently, the variation of re-equilibration timescale between rock-forming minerals and water is coherently large for sample 01TZS06 (Table 2 and the section below).

**Table 2:** Parameters of surface-reaction oxygen exchange.

Sample number	T (°C)*	(W/R)_c_^†^	Quartz					Alkali feldspar				
			Xs^‡^	ρ^§^ (g/cm^3^)	log r^¶^(moles O m^-2^ s^-1^)	a** (cm)	t^††^ (Kyr)	Xs^‡^	ρ^§^ (g/cm^3^)	log r^¶^(moles O m^-2^ s^-1^)	a** (cm)	t^††^ (Kyr)
**Granitoid**				2.66					2.56			
01HP05	140 ± 5	4.49 ± 0.15	0.182 ± 0.005		− 10.18 ± 0.10	0.25	730 ± 105	0.182 ± 0.005		− 8.71 ± 0.08	0.25	20 ± 3
						0.05	145 ± 20				0.05	5 ± 1
02TZ01	375	1.78 ± 0.20	0.362 ± 0.028		− 8.05	0.25	8.0 ± 0.3	0.362 ± 0.028		− 6.99	0.25	0.4 ± 0.0
						0.05	1.5 ± 0.1				0.05	0.1 ± 0.0
**Gneiss**												
01TZS06	130 ± 5	4.85 ± 0.38	0.172 ± 0.011		− 10.32 ± 0.11	0.25	980 ± 190	0.172 ± 0.011		− 8.82 ± 0.09	0.25	30 ± 5
						0.05	195 ± 40				0.05	6 ± 1

*Re-equilibration temperature from Table 1.

^†^Closed system (W/R)_c_ ratio refers to Figs. S1–S3.

^‡^Mole fraction of mineral oxygen re-equilibrated with a water.

^§^Mineral density from Ref.^48^.

^¶^Rate constant after Refs.^39,42^.

**Grain radius.

^††^Time required for attaining 99% oxygen exchange (i.e., F value in the following equation) between a spherical mineral and water. As previously formulated for the closed system^39,42^, ${\text{t}}~ = ~\frac{{ - \ln (1 - {\text{F}}) \times ({\text{W}}/{\text{R}})_{{\text{c}}} \times {\text{X}}_{{\text{s}}} \times {\text{a}} \times \uprho }}{{3 \times \left[ {1 + ({\text{W}}/{\text{R}})_{{\text{c}}} } \right] \times {\text{r}} \times 10^{{ - 4}} }}$, where all variables are listed in Table 2 for corresponding samples.

### The evolution of magmatic water

In spite of the reliability discussed above, the potential causes for the magmatic water with a mildly high ${\updelta }^{18}{\text{O}}_{\text{W}}^{\text{i}}$ value of 2.81 ± 0.05‰ are further accounted for herein. Since the mildly high ${\updelta }^{18}{\text{O}}_{\text{W}}^{\text{i}}$ value is theoretically inverted from the granitoid (Table 1, Fig. 4), it would be an evolved rather than primary magmatic water. There are a couple of pathways for the evolution of magmatic water with the mildly high ${\updelta }^{18}{\text{O}}_{\text{W}}^{\text{i}}$ value.

**Figure 5:**
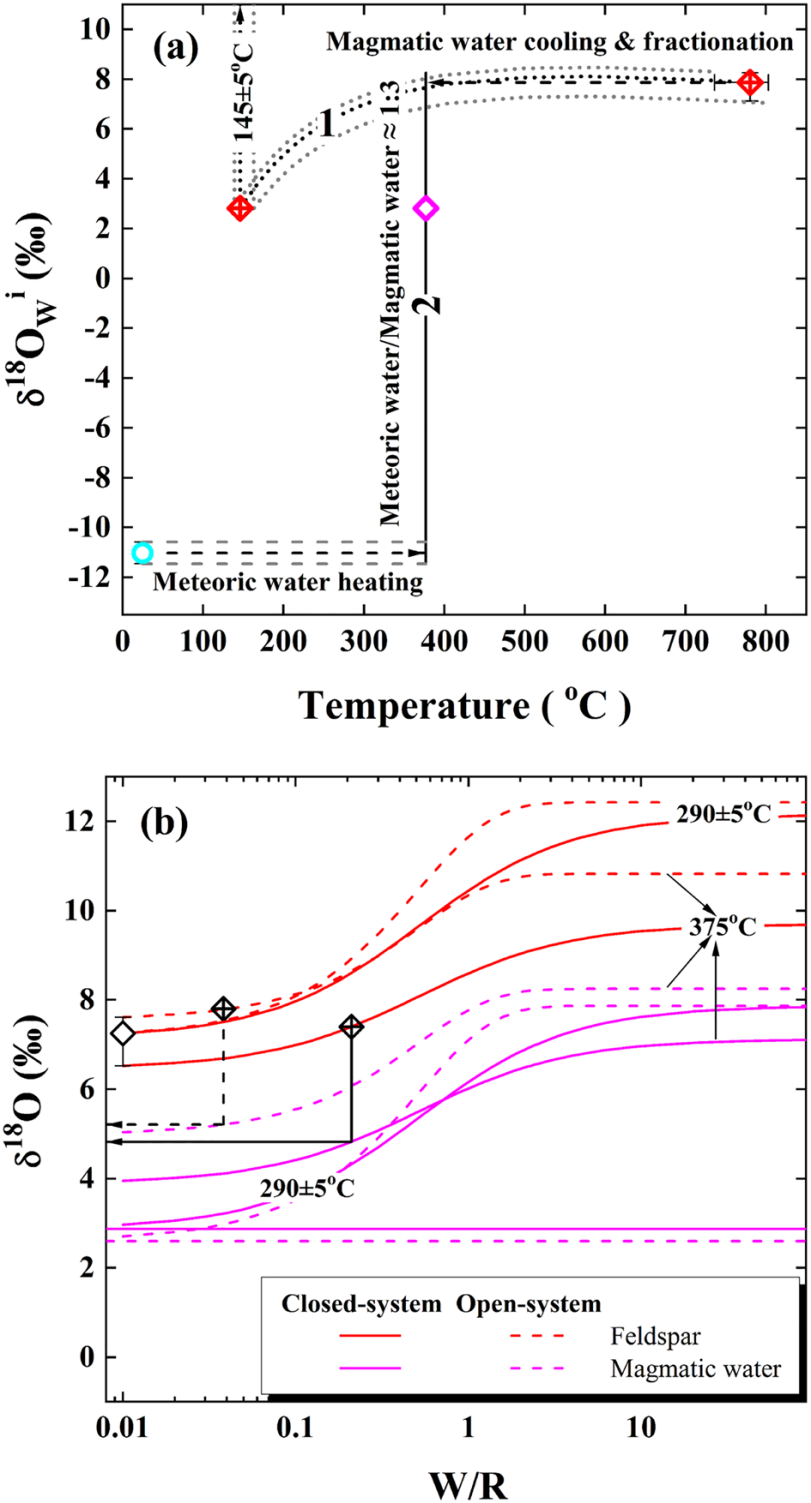
Diagrams of oxygen isotopic evolution. (**a**) Line 1 with gray envelopes denotes thermodynamic fractionation of the primary magmatic water in the course of cooling, whereas line 2 illustrates a binary mixing between the cooled magmatic and heated meteoric water under an isothermal condition. The oxygen isotopes of primary magmatic water are constrained by the observed zircon δ^18^O values of the Tianzhushan granitoid pluton at corresponding magmatic temperatures (Table S1), whereas those of the meteoric water are adopted from sample 01HP05 (Table 1) and reasonably assumed to be applicable on the orogenic scale. (**b**) Final δ^18^O values of alkali feldspar and magmatic water after interacting with each other at labelled temperatures. The initial δ^18^O values of alkali feldspar are calculated with the observed zircon δ^18^O values on the pluton scale, whereas those of primary magmatic water refer to (**a**). Horizontal lines illustrate the maximum variability of ${\updelta }^{18}{\text{O}}_{\text{W}}^{\text{i}}$ values of the evolved magmatic water theoretically inverted from sample 02TZ01 (Table 1, Figs. 4b,e). Data points on the red curves denote the increased δ^18^O values of alkali feldspar observed from the Tianzhushan pluton (Table S1), whereas arrowed lines illustrate the corresponding oxygen isotopes of the magmatic water after exchanging with alkali feldspar.

First, the magmatic water with a mildly high ${\updelta }^{18}{\text{O}}_{\text{W}}^{\text{i}}$ value of 2.81 ± 0.05‰ could in principle evolve from the persistent cooling of a primary magmatic water (curve 1 with error envelopes in Fig. 5a) because of the large fractionation of oxygen isotopes between zircon and water at low temperature. A higher ${\updelta }^{18}{\text{O}}_{\text{W}}^{\text{i}}$ value of 7.68 ± 0.29‰, however, was thermodynamically fractionated when a primary magmatic water was cooled down to the re-equilibration temperature of 375 °C. Apparently, this value is statistically indistinguishable from 7.87 ± 0.27‰ of the primary magmatic water (red symbol point with error bars in Fig. 5a). On the other hand, a temperature of 145 ± 5 °C is hypothetically required in order to further decrease the primary magmatic water down to the mildly high ${\updelta }^{18}{\text{O}}_{\text{W}}^{\text{i}}$ value of 2.81 ± 0.05‰ (arrowed line with error envelopes on the top side in Fig. 5a). The magmatic temperatures of 780 ± 20 °C, however, are well constrained through oxygen isotope fractionation of quartz-zircon pairs for the Tianzhushan granitoid pluton (Fig. 4b). Thereby, the prerequisite temperature of 145 ± 5 °C seems too low to reasonably decrease the ${\updelta }^{18}{\text{O}}_{\text{W}}^{\text{i}}$ value of primary magmatic water down to the mildly high value of 2.81 ± 0.05‰ only via the equilibrium fractionation of oxygen isotopes and is evidently incompatible with the re-equilibration temperature of 375 °C for the studied granitoid during the magma cooling processes. In other words, the magmatic water can persistently cool down even to the room-temperature, yet its ${\updelta }^{18}{\text{O}}_{\text{W}}^{\text{i}}$ value cannot be decreased any more after the constituent minerals were kinetically blocked for oxygen exchanging.

**Figure 6:**
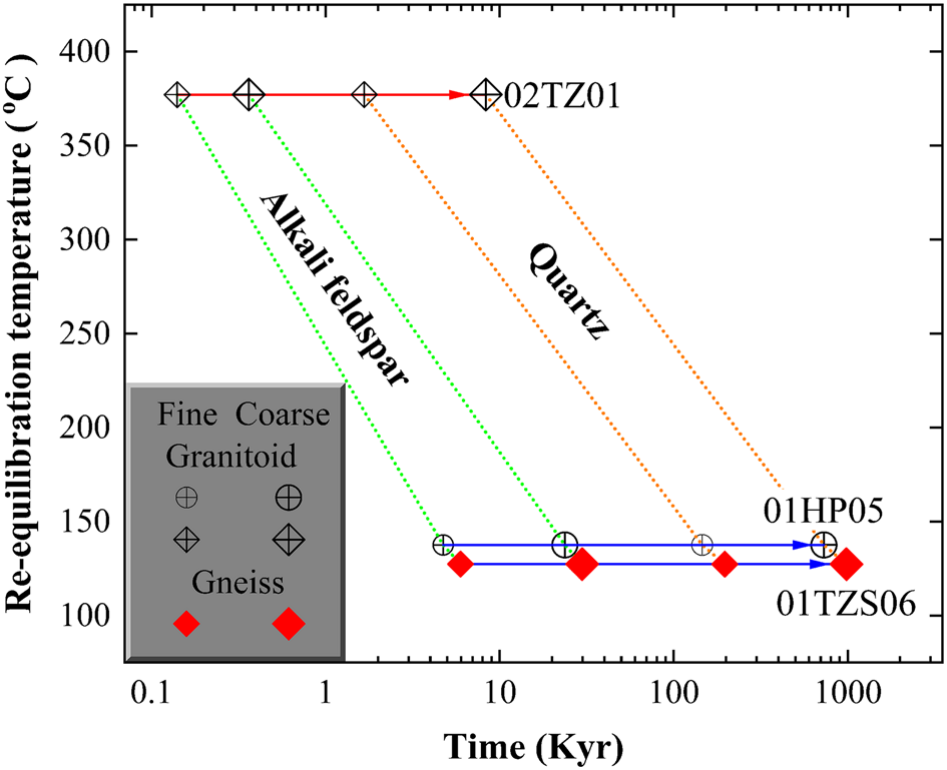
The relationship between time and re-equilibration temperature of fossil hydrothermal systems developed across the Dabie orogen. Symbol points denote mean values of rock-forming minerals with varied grain size sequentially re-equilibrated with the evolved magmatic (sample 02TZ01) or ancient meteoric water (samples 01HP05 and 01TZS06 in Table 2) through the surface-reaction oxygen exchange in the closed systems, respectively. Yet, error bars are almost invisible and omitted herein. Note that log10 scale of X axis is adopted for clarity, other details see text.

Secondly, oxygen isotopes of water and rock can be intrinsically altered both in the course of water–rock interaction as what is so called. Thus, the internal interaction with a host rock could be an alternative to result in the evolved magmatic water with a mildly high ${\updelta }^{18}{\text{O}}_{\text{W}}^{\text{i}}$ value of 2.81 ± 0.05‰. Since the alkali feldspar is kinetically more susceptible to exchange oxygen with water than sluggish quartz and/or inert zircon^38–44^ (also see Fig. S4, Fig. 6, Table 2), the evolution of primary magmatic water is accordingly conducted with alkali feldspar. As shown in Fig. 5b, oxygen isotopes of the primary magmatic water (pink curves) simultaneously decreased with the increases of alkali feldspar (red curves). With the increased δ^18^O values of alkali feldspar observed from the Tianzhushan pluton (Table S1), oxygen isotopes of the primary magmatic water correspondingly decreased after the subsolidus exchange with alkali feldspar at 375 °C (arrowed lines in Fig. 5b). The resulting oxygen isotopes from 4.82 to 5.21‰, however, are apparently higher than 2.81 ± 0.05‰ theoretically inverted for the evolved magmatic water. While the mildly high ${\updelta }^{18}{\text{O}}_{\text{W}}^{\text{i}}$ value of 2.81 ± 0.05‰ could ultimately approach at a lower re-equilibration temperature of 290 ± 5 °C, an extremely low or unreasonable W/R ratio is yielded (labelled curves in Fig. 5b). Energetically, this cooled down magmatic water has to be heated up to the temperature of 375 °C again in order to repeatedly re-equilibrate with the studied granitoid. This thermally down-and-up looped process seems less realistic although it cannot be fully discarded.

Therefore, the evolved magmatic water with a mildly high ${\updelta }^{18}{\text{O}}_{\text{W}}^{\text{i}}$ value of 2.81 ± 0.05‰ cannot be reasonably addressed with the persistent cooling of a primary magmatic water alone or the post-magmatic autometasomatism. In this case, the dilution of the ${\updelta }^{18}{\text{O}}_{\text{W}}^{\text{i}}$ value could be more likely by a parcel inflowing of light water depleted in ^18^O. While the ancient meteoric water with low ${\updelta }^{18}{\text{O}}_{\text{W}}^{\text{i}}$ values is theoretically inverted from both samples 01HP05 and 01TZS06 (Table 1), the ${\updelta }^{18}{\text{O}}_{\text{W}}^{\text{i}}$ value of − 11.01 ± 0.43‰ is temporally more appropriate for admixing with the primary magmatic water of the early Cretaceous post-collisional magmatism. An input around 25 ± 2 wt% of this ancient meteoric water can thus dilute  the ${\updelta }^{18}{\text{O}}_{\text{W}}^{\text{i}}$ value of a primary magmatic water down to 2.81 ± 0.05‰ through a binary mixing process under an isothermal condition of 375 °C (line 2 with symbol point in Fig. 5a). This evolved magmatic water was then re-equilibrated with and concurrently lowered oxygen isotopes of rock-forming minerals of the studied granitoid during the post-magmatic hydrothermal processes. Because the meteoric water could become concentrated in ^18^O as it travelled along its flow-path downwards under the condition with a low W/R ratio, a systematically more input of this modified meteoric water by the time it mixing with the primary magmatic water would be expected. However, W/R ratios quantified for the external infiltration of the ancient meteoric water are generally over 1.0 herein (Figs. S1, S2, Fig. 3), this ^18^O-concentrated process thus seems a less real scenario.

### Kinetic modelling of oxygen exchange

How did oxygen exchange between mineral and water although ${\updelta }^{18}{\text{O}}_{\text{W}}^{\text{i}}$ values of ancient meteoric and evolved magmatic water are theoretically inverted from hydrothermally altered minerals above? Was the lifetime of fossil hydrothermal systems developed across the Dabie orogen geologically reasonable? These issues are further discussed below.

#### Diffusive oxygen exchange

As an elementary process, diffusion plays one of essential roles for oxygen exchange between mineral and water. Because oxygen diffusivity of zircon is systematically lower than that of quartz and alkali feldspar under similar conditions (Fig. S4a), zircon is thus one of the most resistant accessory minerals to hydrothermal alteration and can faithfully retain its original δ^18^O value^41,43,44^. Disequilibria of oxygen isotopes accordingly appear between the resistant and susceptible minerals during short-lived hydrothermal processes (Fig. 2), in qualitative accordance with their kinetic behaviours.

On the other hand, it has been well known that the diffusion is thermally activated (cf., Arrhenius relationship in caption of Fig. S4a). Hereby, the timescale of diffusive oxygen exchange between constituent minerals and water is quantified at corresponding re-equilibration temperatures with the model of spherical minerals herein (see caption of Fig. S4b). It can be seen that re-equilibration temperatures are overall less than 400 °C in this study (Table 1). Since oxygen diffusion rate of α-quartz is rather slow at this temperature interval (i.e., D value in Fig. S4a), it is therefore too long to reset its δ^18^O value via diffusive oxygen exchange with water. For example, an unreasonable timescale of 60 Myr is yielded for the sluggish α-quartz even with a fine-grained size at the high-end of re-equilibration temperatures (arrowed dashed line on the left side in Fig. S4b). While a timespan of no less than 20 thousand years (Kyr) is kinetically quantified for the susceptible alkali feldspar to diffusively lower its oxygen isotopes under similar conditions (arrowed solid line on the left side), a duration up to 120 billion years (Gyr) is unrealistically required for the coarse-grained alkali feldspar at the low-end of re-equilibration temperatures (arrowed line on the right side). Thus, these quantitatively suggest that diffusion is a less likely mechanism for oxygen exchange in this study.

#### Surface-reaction oxygen exchange

Compared to diffusive processes, oxygen exchange rates of the surface-reaction between rock-forming minerals and water are several orders of magnitude high (Refs.^39,42^ and r values in Table 2). Given that thermodynamic re-equilibration was achieved and/or reproduced between quartz and alkali feldspar oxygen isotopes for the studied samples (Figs. S1–S3 and labelled data points in Fig. 3), this probably suggests that surface-reaction instead of volume diffusion eventually modulated oxygen exchange herein. Mechanisms of the surface-reaction like dissolution, reprecipitation and exchange along micro-fractures and/or within networks were proposed to account for the varied quartz and/or alkali feldspar oxygen isotopes during hydrothermal processes^39,42,45–47^.

Based on parameters listed in Table 2, the time for attaining 99% oxygen exchange via the surface-reaction between rock-forming minerals and water was accordingly calculated. Since the oxygen exchange rate between alkali feldspar and water is more rapid than that between quartz and water^39,42^, thermodynamic re-equilibration with water was readily approached for the fine-grained alkali feldspar at the temperature interval throughout this study (Table 2, Fig. 6). Then, a longer timescale was systematically yielded for the coarse-grained quartz.

Owing to the relatively high re-equilibration temperature of 375 °C for sample 02TZ01, a short duration from 0.1 ± 0.0 to 8.0 ± 0.3 Kyr is hereby quantified for alkali feldspar and quartz sequentially re-equilibrated with the evolved magmatic water, respectively. As an internally buffered process, the paradoxical lowering and rapid re-equilibrating of oxygen isotopes between rock-forming minerals and the evolved magmatic water could occur within the deep or inner portion of the Tianzhushan granitoid pluton. For the gneissic country rock intruded by the Tianzhushan pluton, a maximum timespan was shifted to 980 ± 190 Kyr at 130 ± 5 °C for the shallow infiltration of ancient meteoric water during exhumation processes of the retrograde metamorphism (sample 01TZS06 in Table 2, Fig. 6). Nonetheless, the lifetime of fossil hydrothermal systems developed across the Dabie orogen in central-eastern China could be less than 1.2 Myr for the concurrently lowered oxygen isotopes of hydrothermally altered rock-forming minerals by the ancient meteoric water with low ${\updelta }^{18}{\text{O}}_{\text{W}}^{\text{i}}$ values from − 11.01 ± 0.43 to − 8.52 ± 0.56‰ or an evolved magmatic water with mildly high ${\updelta }^{18}{\text{O}}_{\text{W}}^{\text{i}}$ value of 2.81 ± 0.05‰, respectively.

## Methods

### Geological context and sampling

As previously summarised by Wei and Zhao^16,17^, the Dabie-Sulu orogen is characterised by the largest occurrence of the microdiamond- and/or coesite-bearing UHP metamorphic rocks worldwide^49–52^. Triassic ages of 200 to 240 Ma were dated with distinctive geochronometers for the eclogite-facies rocks and their cooling histories during exhumation processes were accordingly quantified^27–33^. Furthermore, the ultrahigh ε_Nd_(t) value up to + 264 ever measured for eclogites^53^ and zircons with the reported lowest δ^18^O values down to about − 11‰ were found^23–25,54^.

Compared with the sporadically outcropped lenses or blocks of UHP eclogites, composite plutons and batholiths of the early Cretaceous post-collisional granitoid are the predominant igneous rocks although a number of coeval small mafic to ultramafic plutons have been documented in the Dabie orogen^18–20,55–67^. Most plutons and batholiths were dated with zircon U–Pb techniques and the early Cretaceous ages ranging from 125 to 135 Ma were therefore yielded. The affinity with the South China Block was further indicated by the upper intercept age of Neoproterozoic for available zircon U–Pb datings. The petrogenetic linkage of the early Cretaceous post-collisional igneous rocks to an old enriched source(s) was evidenced by zircon Hf and whole-rock Nd–Sr isotopes.

It can be seen in Fig. 1 that the studied granitoids and their gneissic country rocks mainly occupy the northern and central-eastern lithotectonic units of the DBB. From north to south, the Hepeng pluton (HP) lies in the utmost eastern tip of the Northern Huaiyang volcanic-sedimentary belt, whereas the Tianzhushan pluton (TZS) is adjacent to the Central Dabie UHP metamorphic belt, respectively. The intrusive contact between granitoids and gneissic country rocks was unambiguously observed in the field. For comparison, two gneisses from the Sidaohe without being intruded by granitoids in the Hong’an Block are also studied.

Since all of samples were collected from quarries and/or along road cuttings, medium-grained granitoids and gneisses are thus less weathered and/or fresh. Petrographically, quartz, feldspar, biotite and sometimes amphibole are major rock-forming minerals. Accessory minerals include zircon but magnetite is occasionally present.

### Analysis of oxygen isotopes

First, zircon, quartz and feldspar were separated and concentrated from whole-rocks through the conventional crushing, gravimetric, heavy liquid and magnetic techniques. Then, the separated zircons were sequentially treated with concentrated HCl, HNO_3_ and HF acids under room conditions overnight to remove metamict zircons and any other impurities. The purity of mineral separates is generally better than 98% with optical microscope examination.

Oxygen isotopes were analysed with the laser fluorination online techniques^68,69^, and an air-lock chamber was employed to avoid the “cross-talk” of reactive alkali feldspar^70^. The conventional δ^18^O notation in permil (‰) relative to Vienna Standard Mean Ocean Water (VSMOW) is reported in Table S1.

The garnet standard, UWG-2, was routinely analysed to control the quality of δ^18^O analyses. The daily average of measured δ^18^O values of UWG-2 varied from 5.54 to 5.89‰ for 15 analytical days over three months, and the daily analytical precision is better than ± 0.11‰. In terms of the accepted UWG-2 value of 5.80‰, raw δ^18^O values of mineral separates were accordingly corrected. The international standard (NBS 28 quartz) was analysed during the course of this study, and the corrected δ^18^O values for NBS 28 are from 9.31 to 9.69‰.

The reproducibility of fresh crystalline zircon δ^18^O analyses is excellent throughout this study. As shown in Table S1, the 1SD of most duplicate measurements with one triplicate is less than ± 0.05‰, which is within the maximum routine analytical errors demonstrated by daily UWG-2 garnet standard measurements.

### Theoretical inversion of ${\updelta }^{18}{\text{O}}_{\text{W}}^{\text{i}}$ value

For constituent minerals achieved thermodynamic re-equilibrations with water, the ${\updelta }^{18}{\text{O}}_{\text{W}}^{\text{i}}$ value can be theoretically inverted^16,17^. For the closed system, two equations were derived for alkali feldspar (Ksp) and quartz (Qtz), respectively:

1 $${\delta^{18}} {\text{O}_{{{\text{Ksp}}}}^{{\text{f}}}} = \frac{{\delta^{{18}} {\text{O}_{{{\text{Ksp}}}}^{{\text{i}}}} +\left[ {\delta ^{{18}} {\text{O}_{{\text{W}}}^{{\text{i}}}} + ({\Delta^{{18}}} {\text{O}_{{\text{W}}}^{{{\text{Ksp}}}}} )_{{\text{r}}} } \right] \boldsymbol{\cdot} \left( {{\text{W}}/{\text{R}}} \right)_{{\text{c}}} \boldsymbol{\cdot} \left( {{\text{n}}_{{\text{W}}}^{{\text{O}}} /{\text{n}}_{{{\text{Ksp}}}}^{{\text{O}}} } \right)}}{{1 +\left( {{\text{W}}/{\text{R}}} \right)_{{\text{c}}} \boldsymbol{\cdot} \left( {{\text{n}_{{\text{W}}}^{{\text{O}}}} /{\text{n}_{{{\text{Ksp}}}}^{{\text{O}}} }} \right)}}$$

2 $${\updelta }^{18}{\text{O}}_{\text{Qtz}}^{\text{f}} = \frac{{\updelta }^{18}{\text{O}}_{\text{Qtz}}^{\text{i}} + \left[{{\updelta }^{18}{\text{O}}_{\text{W}}^{\text{i}} + {{\text{(}\Delta}^{18}{\text{O}}}_{\text{W}}^{\text{Qtz}}\text{)}}_{\text{r}}\right] \boldsymbol{\cdot}{\left({\text{W}}/{\text{R}}\right)}_{\text{c}} \boldsymbol{\cdot} \left({\text{n}}_{\text{W}}^{\text{O}}/{\text{n}}_{\text{Qtz}}^{\text{O}}\right)}{\text{1} + {\left({\text{W}}/{\text{R}}\right)}_{\text{c}} \boldsymbol{\cdot} \left({\text{n}}_{\text{W}}^{\text{O}}/{\text{n}}_{\text{Qtz}}^{\text{O}}\right)}\text{}$$

3 $$\text{and }{\updelta }^{18}{\text{O}}_{\text{Ksp}}^{\text{i}} = {\updelta }^{18}{\text{O}}_{\text{Zrc}}^{\text{i}} + \text{ } {{{\text{(}}\Delta^{18}{\text{O}}}_{\text{Zrc}}^{\text{Ksp}}\text{)}}_{\text{m}}\text{ or }{\updelta }^{18}{\text{O}}_{\text{Qtz}}^{\text{i}} = {\updelta }^{18}{\text{O}}_{\text{Zrc}}^{\text{i}} + {{{\text{ (}\Delta}^{18}{\text{O}}}_{\text{Zrc}}^{\text{Qtz}}\text{)}}_{\text{m}}\text{}$$

where ${\updelta }^{18}{\text{O}}_{\text{Ksp}}^{\text{f}}$ and ${\updelta }^{18}{\text{O}}_{\text{Qtz}}^{\text{f}}$ are final values observed for specified minerals; ${\updelta }^{18}{\text{O}}_{\text{Ksp}}^{\text{i}}$ and ${\updelta }^{18}{\text{O}}_{\text{Qtz}}^{\text{i}}$ values can be calculated by Eq. (3) with the observed zircon (Zrc) δ^18^O values at magmatic or metamorphic temperature (${{{\text{i.e., (}\Delta}^{18}{\text{O}}}_{\text{Zrc}}^{\text{Ksp}}\text{)}}_{\text{m}}{\text{or}} {{{\text{ (}\Delta}^{18}{\text{O}}}_{\text{Zrc}}^{\text{Qtz}}\text{)}}_{\text{m}}{\text{value}}$); and ${{{\text{(}\Delta}^{18}{\text{O}}}_{\text{W}}^{\text{Ksp}}\text{)}}_{\text{r}}$ or ${{{\text{(}\Delta}^{18}{\text{O}}}_{\text{W}}^{\text{Qtz}}\text{)}}_{\text{r}}$ value can be calculated with the re-equilibration temperature. Moreover, ${{\text{n}}_{\text{W}}^{\text{O}}}/{{\text{n}}_{\text{Ksp}}^{\text{O}}}\,{{\text{and}}} \, {{\text{n}}_{\text{W}}^{\text{O}}}/{{\text{n}}_{\text{Qtz}}^{\text{O}}}$ ratios are actually constants between water and alkali feldspar as well as quartz (last row in Table 1). In this case, both ${\updelta}{^{18}{\text{O}}}_{\text{W}}^{\text{i}}$ value and ${\left({\text{W}}/{\text{R}}\right)}_{\text{c}}$ ratio can thus be solved by combining Eqs. (1) and (2).

In order to be self-consistent, theoretically calculated oxygen isotope fractionations at temperatures ranging from 0 to 1200 °C are adopted throughout this study^21^. Because the discrepancy between theoretical calculation and experimental calibration or empirical estimation is not remarkable for oxygen isotope fractionations of the studied constituent minerals^71–74^, this will not considerably influence our results herein.

For the open system, a similar inverse procedure can be applied. Due to the term of natural logarithm or exponential function (i.e., (W/R)_o_ = ln[(W/R)_c_ + 1]), an analytical expression cannot be obtained. Under this circumstance, the numerical reiteration with a goal precision of at least ± 0.0001 is conducted to theoretically invert the ${\updelta }^{18}{\text{O}}_{\text{W}}^{\text{i}}$ value and (W/R)_o_ ratio in this study.

## Supplementary Information

Supplementary Information.

## Data Availability

The authors declare that all relevant data are available within the article and its Supplementary Information Files.
