# Supplementary material for: Paradoxically lowered oxygen isotopes of hydrothermally altered minerals by an evolved magmatic water
Source: Sci Rep. 2022 Sep 28;12:16213. doi: 10.1038/s41598-022-19921-y (PMC9519577; doi:10.1038/s41598-022-19921-y)
Supplement: Supplementary file 1 — Supplementary Information. [file 41598_2022_19921_MOESM1_ESM.pdf]

Supplementary Information for

Paradoxically lowered oxygen isotopes of hydrothermally altered minerals by an evolved magmatic water

Chun-Sheng Wei✉ **& Zi-Fu Zhao**

CAS Key Laboratory of Crust-Mantle Materials and Environments, School of Earth and Space Sciences, University of Science and Technology of China, Hefei 230026, China.

✉email: wchs@ustc.edu.cn

This PDF file includes:

Figs. S1–S4

Table S1

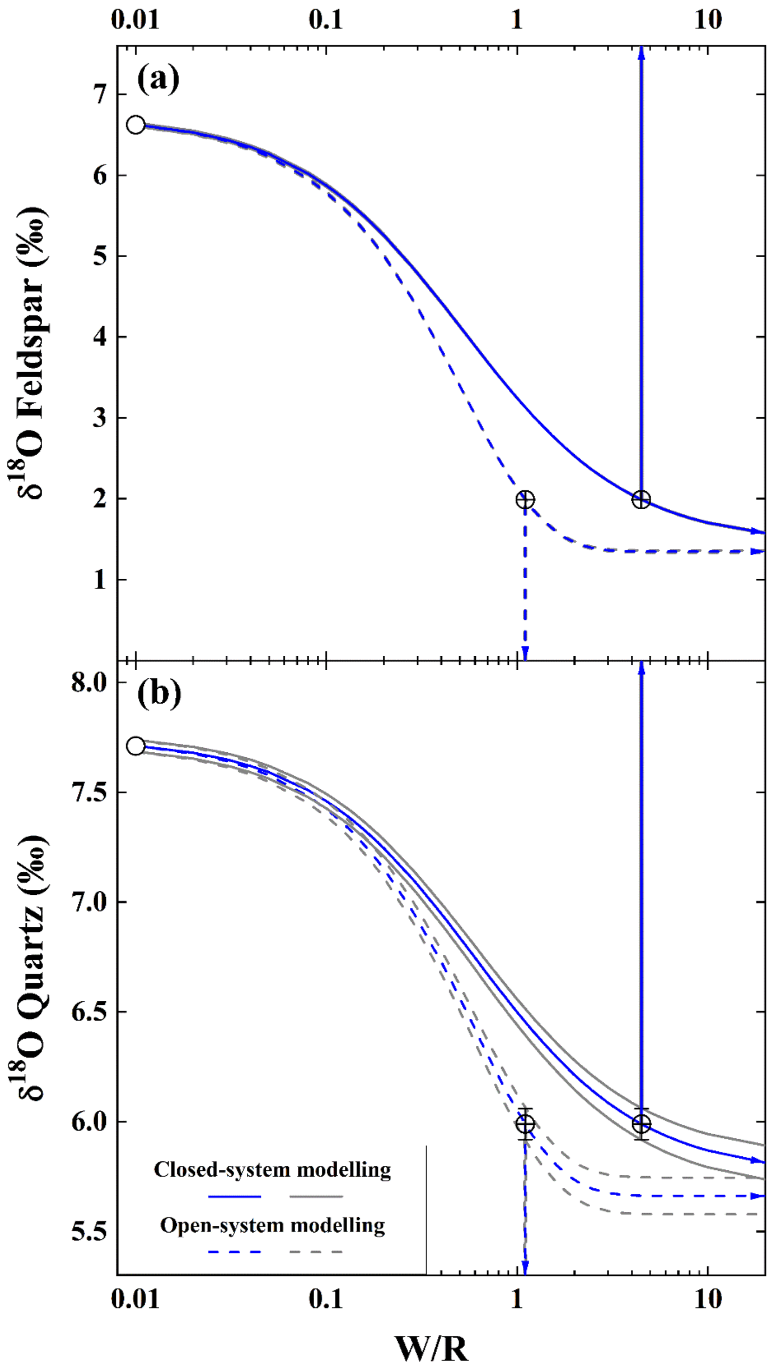

Figure S1. The concurrent lowering of oxygen isotopes with W/R ratios for alkali feldspar (**a**) and quartz (**b**) by the meteoric water for sample 01HP05 from the early Cretaceous post-collisional Hepeng granitoid pluton. Due to the limited variability of the observed and initial oxygen isotopes, the gray envelopes are almost invisible for alkali feldspar in (**a**). Arrowed vertical lines illustrate W/R ratios required to reproduce the observed $\delta^{18}O$ values. Note that log10 scale of X axes and different scales of Y axes in (**a**) and (**b**) are adopted for clarity. Other details refer to Fig. 2.

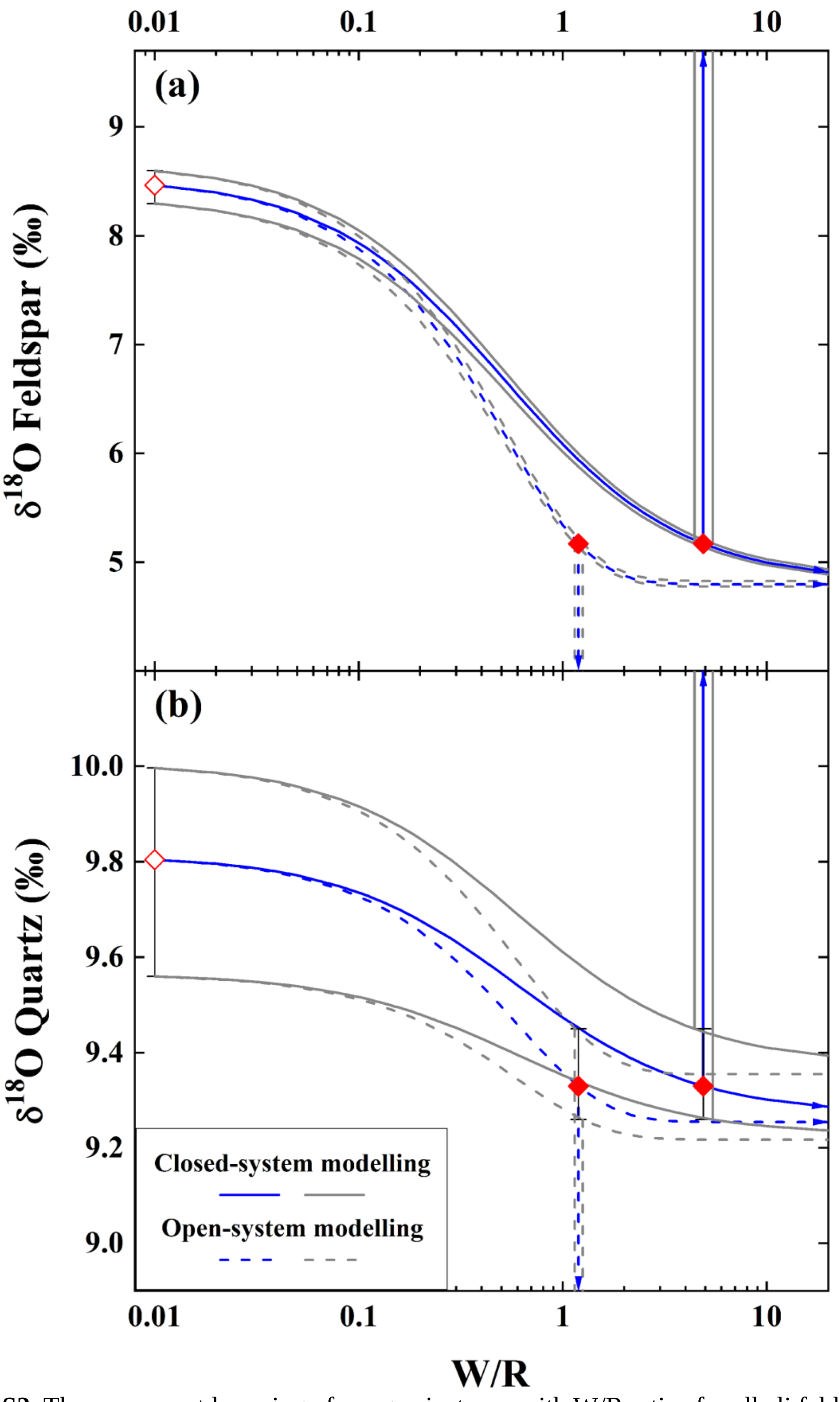

Figure S2. The concurrent lowering of oxygen isotopes with W/R ratios for alkali feldspar (**a**) and quartz (**b**) by the meteoric water for sample 01TZS06 from the Triassic gneissic country rock intruded by the Tianzhushan pluton.

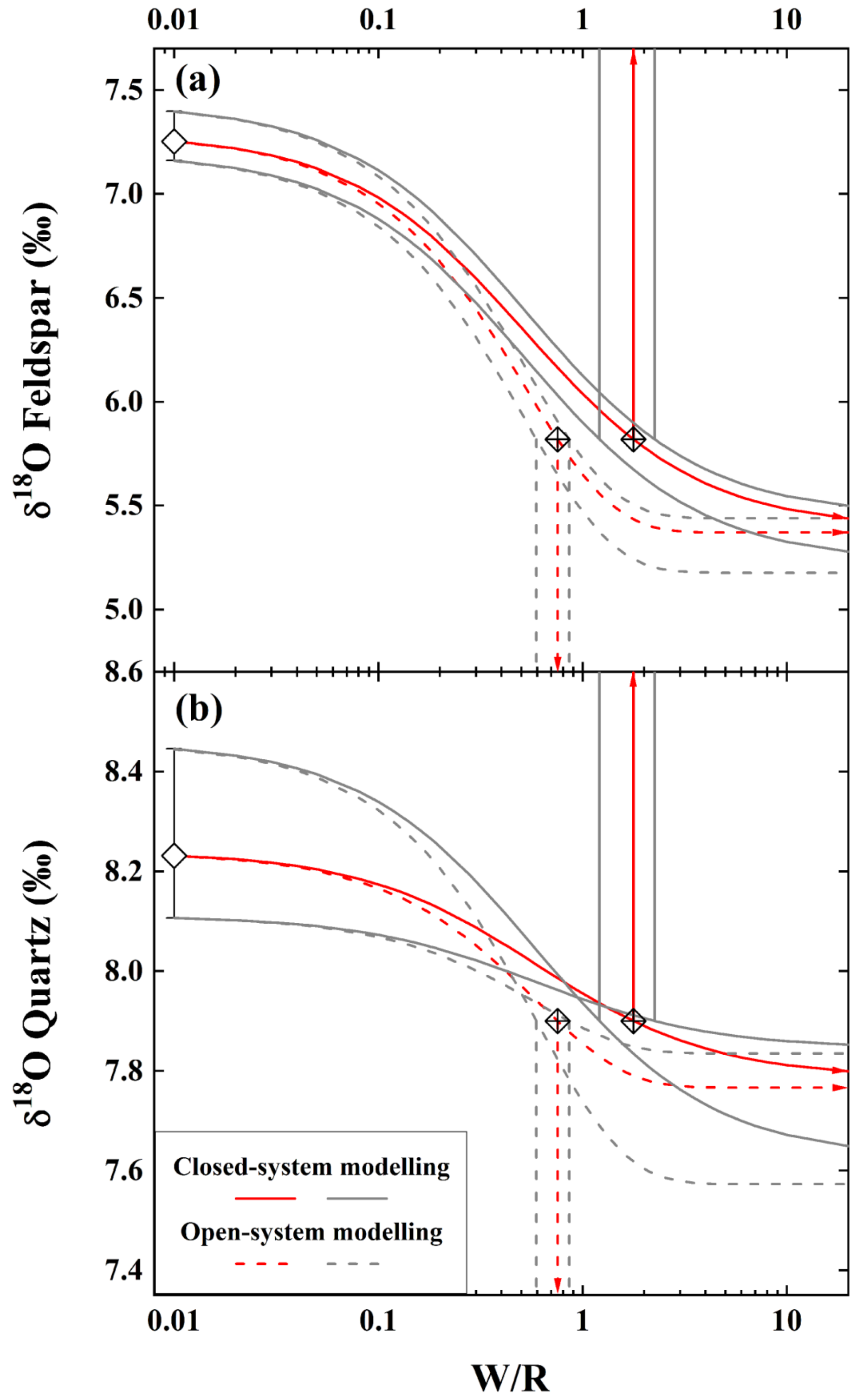

Figure S3. The concurrent lowering of oxygen isotopes with W/R ratios for alkali feldspar (**a**) and quartz (**b**) by the evolved magmatic water for sample 02TZ01 from the early Cretaceous post-collisional Tianzhushan granitoid pluton.

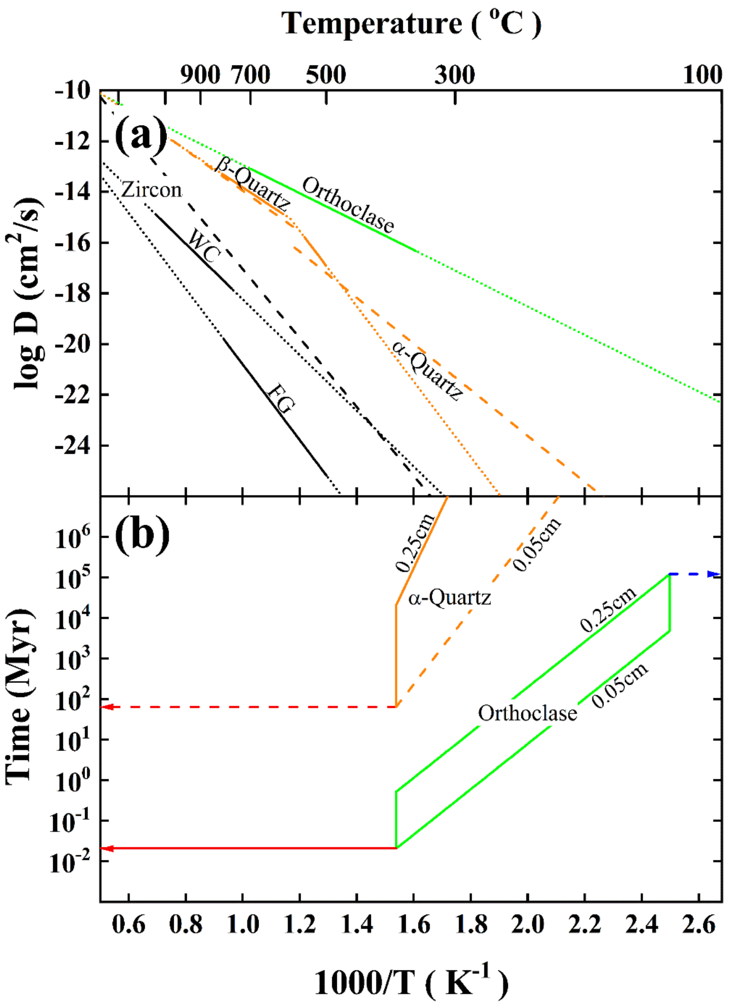

Figure S4. Modelling of diffusive oxygen exchange. (**a**) Arrhenius plot of oxygen diffusion within minerals under wet conditions ($D = D_0 \times e^{E_a/RT}$, where D_0 is pre-exponential factor, E_a is activation energy, R is gas constant and T is thermodynamic temperature in Kelvin, respectively). Dashed lines are theoretical calculations[44], whereas dotted lines with solid segments are experimental determinations. For zircon, WC is from ref.[43] and FG is from ref.[41], respectively. Quartz data are from ref.[40], and those of orthoclase are after ref.[38]. (**b**) Oxygen diffusive modelling for α-quartz and alkali feldspar with two radii (solid lines based on experimentally determined parameters whereas dashed line on theoretical ones). Model of spherical minerals is adopted ($D \times t/a^2 = 0.03$, where D is diffusion rate, t is time and a is grain size for the specified mineral, respectively), and arrowed lines denote the timescale of rock-forming minerals diffusively exchanged oxygen with meteoric (blue) or evolved magmatic water (red) in this study. Other details see text.

Sample number	δ18O Zircon (‰)			δ18O Quartz (‰)			δ18O Alkali feldspar (‰)			GPS data
	Measured	Ave	1SD	Measured	Ave	1SD	Measured	Ave	1SD	
**Hepeng pluton (HP)**[†]										
**Granitoid**[‡]										
01HP04	4.55, 4.54	4.55	0.01	7.69	7.69	/	6.42	6.42	/	31º13´37″, 116º45´25″
01HP05	4.64, 4.60	4.62	0.03	5.92, 6.06	5.99	0.10	1.99	1.99	/	31º12´42″, 116º47´28″
**Sidaohe (SDH)**										
**Gneiss**[‡]										
00DB63	-0.59, -0.67, -0.64	-0.63	0.04	3.11, 2.91	3.01	0.14	1.77	1.77	/	31º22´12″, 115º04´09″
00DB64	-1.75, -1.51	-1.63	0.17	2.53, 2.51	2.52	0.01	1.22	1.22	/	31º22´12″, 115º04´09″
**Tianzhushan/Yuexi pluton (TZS)**										
**Granitoid**[§]										
03TZ01	4.98	4.98	/	8.14	8.14	/	7.00	7.00	/	30º50´41″, 116º17´14″
03TZ02	5.18	5.18	/	8.01	8.01	/	7.08	7.08	/	30º50´31″, 116º18´09″
03TZ03	5.57	5.57	/	8.53	8.53	/	5.99	5.99	/	30º50´39″, 116º19´05″
03TZ05	5.83	5.83	/	9.16	9.16	/	7.80	7.80	/	30º48´25″, 116º20´45″

03TZ06	5.54	5.54	/	/	/	/	3.76	3.76	/	30º46´41″, 116º20´45″
03TZ08	5.56	5.56	/	8.50	8.50	/	2.14	2.14	/	30º45´42″, 116º20´34″
03TZ09	5.44	5.44	/	8.81	8.81	/	7.40	7.40	/	30º44´20″, 116º22´02″
03TZ10	5.14	5.14	/	7.95	7.95	/	6.50	6.50	/	30º43´25″, 116º23´08″
03TZ11	5.40	5.40	/	8.33	8.33	/	6.69	6.69	/	30º43´23″, 116º26´52″
03TZ12	5.08	5.08	/	7.93	7.93	/	6.27	6.27	/	30º45´22″, 116º26´06″
03TZ16	4.41	4.41	/	7.48	7.48	/	4.18	4.18	/	30º43´51″, 116º28´02″
03TZ17	5.09	5.09	/	7.98	7.98	/	6.44	6.44	/	30º44´26″, 116º27´11″
03TZ18	4.94	4.94	/	7.85	7.85	/	6.47	6.47	/	30º44´35″, 116º27´07″
03TZ19	5.47	5.47	/	8.28	8.28	/	6.22	6.22	/	30º44´33″, 116º27´27″
03TZ20	5.29	5.29	/	8.17	8.17	/	6.10	6.10	/	30º43´56″, 116º27´26″
03TZ22	5.44	5.44	/	8.26	8.26	/	7.58	7.58	/	30º44´46″, 116º29´08″
02TZ01	5.32	5.32	/	7.90	7.90	/	5.82	5.82	/	30º43´45″, 116º26´49″
02TZ02	5.37	5.37	/	8.36	8.36	/	6.70	6.70	/	30º43´40″, 116º26´47″
02TZ03	5.17	5.17	/	8.18	8.18	/	6.02	6.02	/	30º43´28″, 116º26´53″
02TZ04	5.37	5.37	/	8.26	8.26	/	3.35	3.35	/	30º43´22″, 116º27´19″

02TZ05	5.00	5.00	/	7.96	7.96	/	2.32	2.32	/	30º43´38″, 116º27´47″
**Gneiss**[‡]										
01TZS06	5.87, 5.88	5.88	0.01	9.45, 9.26, 9.27	9.33	0.11	5.17	5.17	/	30º42´55″, 116º27´48″
01TZS07	-3.78, -3.71	-3.75	0.05	0.29, 0.14, 0.34	0.26	0.10	-0.26, -0.01	-0.14	0.18	30º42´06″, 116º29´13″

Table S1. Oxygen isotopes of granitoids and gneisses from the Dabie orogen in central-eastern China[*]. [*]Plutons are alphabetically tabulated throughout this study. [†]Abbreviation within parenthesis is labelled in Fig. 1, and that after / denotes alternative name adopted by other authors. [‡]Data from refs.[16,17]. [§]Data after refs.[60,62].
